# Detection of Road Potholes by Applying Convolutional Neural Network Method Based on Road Vibration Data

**DOI:** 10.3390/s23229023

**Published:** 2023-11-07

**Authors:** Furkan Ozoglu, Türkay Gökgöz

**Affiliations:** Department of Geomatic Engineering, Yildiz Technical University, 34349 Istanbul, Turkey; gokgoz@yildiz.edu.tr

**Keywords:** road pothole detection, deep learning, convolution neural network, smartphone sensors, mobile application

## Abstract

In the context of road transportation, detecting road surface irregularities, particularly potholes, is of paramount importance due to their implications for driving comfort, transportation costs, and potential accidents. This study presents the development of a system for pothole detection using vibration sensors and the Global Positioning System (GPS) integrated within smartphones, without the need for additional onboard devices in vehicles incurring extra costs. In the realm of vibration-based road anomaly detection, a novel approach employing convolutional neural networks (CNNs) is introduced, breaking new ground in this field. An iOS-based application was designed for the acquisition and transmission of road vibration data using the built-in three-axis accelerometer and gyroscope of smartphones. Analog road data were transformed into pixel-based visuals, and various CNN models with different layer configurations were developed. The CNN models achieved a commendable accuracy rate of 93.24% and a low loss value of 0.2948 during validation, demonstrating their effectiveness in pothole detection. To evaluate the performance further, a two-stage validation process was conducted. In the first stage, the potholes along predefined routes were classified based on the labeled results generated by the CNN model. In the second stage, observations and detections during the field study were used to identify road potholes along the same routes. Supported by the field study results, the proposed method successfully detected road potholes with an accuracy ranging from 80% to 87%, depending on the specific route.

## 1. Introduction

The increasing urban population has led to a significant rise in the number of vehicles on the roads, resulting in various road surface irregularities, with road potholes being a significant concern. In Turkey, according to the 2022 data from the Turkish Statistical Institute (TUIK), 5229 people lost their lives in traffic accidents, and 288,696 people were injured, with approximately 2.1% of these accidents being attributed to vehicles and around 0.4% to road factors [1]. Although a small fraction of accidents is directly linked to road conditions, the impact is substantial, considering the loss of life and injuries involved. Road potholes alone are reported to contribute to about 50% of accidents caused by road defects (see Table 15 in [2]). Furthermore, road potholes can have adverse effects on vehicles, causing deformations in tires and suspensions. Some vehicle defects leading to traffic accidents may also be attributed to road potholes (see Table 16 in [2]). Road irregularities not only challenge drivers during transportation but also negatively affect the travel experience for passengers, including those who are ill, pregnant, or elderly. Therefore, early detection of road defects/potholes is crucial, both for reducing fatalities, injuries, and financial losses caused by traffic accidents and for ensuring balanced vehicle operation and improved travel quality.

In the literature, various studies have been conducted to assess road conditions automatically for identifying road potholes and other road irregularities. These studies can generally be categorized into two main approaches. Firstly, there are vision-based approaches that employ various imaging methods to capture road defects, including potholes, through image processing analysis [3]. Secondly, there are vibration-based approaches where various types of motion sensors collect vehicle vibration data to detect road anomalies, involving the use of thresholding, machine learning (ML), and dynamic time warping (DTW) techniques [4].

In 3D reconstruction studies within the scope of road imaging, road data, including pavement distress, have been collected by automatically identifying road surface irregularities using cameras and laser scanners [5]. Additionally, road conditions have been assessed through stereovision cameras and laser scanners, resulting in a 3D model of road defects [6]. In contrast to the high cost of the first two methods, low-cost kinetic sensors, such as Kinect sensors, have been used to obtain 3D road pavement distress data [7]. Moreover, some studies based on road imaging methods have been transformed into commercial products like Automatic Road Analyzer (ARAN) [8] and Road Measurement Data Acquisition System (ROMDAS) [9], which are used for road quality assessment.

Regarding pothole detection studies in the vision-based approach, potholes have been detected using 2D image processing techniques, spectral clustering, and color images [10]. Furthermore, color images cropped from high-speed camera videos have been used to automatically segment potholes [11,12]. High-speed 3D reconstruction has provided real-time and cost-effective 3D road defect data [13], and an LiDAR-based pavement distress detection method has been proposed [14], extracting the 3D road surface using a combination of 3D point cloud modeling and 2D image processing algorithms [15]. In addition, some studies based on road imaging methods have employed image detection using support vector machines (SVM) [16] and deep convolutional neural networks (DCNN) [17,18] to identify road cracks.

Using smartphone sensors, pothole detection has been performed based on thresholding approaches [19,20], machine learning techniques [21], data mining algorithms [22], support vector machine (SVM) methods [23], dynamic time warping (DTW) techniques [24], and machine learning (ML) with a sensor-equipped vehicle [25] to monitor road surfaces and detect potholes. When examining the studies on road surface monitoring and pothole detection, it can be observed that deep learning (DL) methods with deep-learning-based image processing have been employed [17,18] in vision-based studies. However, considering vibration-based studies, it is observed that machine learning (ML) methods [23,25] and artificial neural networks (ANN) [26] are predominantly utilized.

A method for recognizing time series data composed of accelerometer signals from the DeepSense framework was proposed [27]. The results of the proposed method in the study were also compared with CNN. DeepSense was recommended as a method that integrates CNN and recurrent neural network (RNN) to leverage local interactions among similar mobile sensors, adapted for applications focusing on time series data processing in mobile sensing [28]. As seen, in the first study aiming to recognize road surfaces using deep learning, integrated convolutional and recurrent layers that utilize data in a time series format were used.

In the literature, it is observed that convolutional layers are used in the context of road surface recognition, particularly when vibration-based data are evaluated within time series. However, no CNN study has been found that transforms road vibration data into pixels. In this study, a novel method was developed to detect road potholes using convolutional neural network techniques by transforming vibration-based data obtained from smartphone sensors into pixels.

To demonstrate the originality of this study, a detailed review of studies that employ vehicle-generated road vibration data to detect road potholes along a road route is provided in Section 2. The methodology used, including data collection, preparation, and processing of vibration data collected from smartphones along the route, is presented in Section 3. Section 4 outlines the development, training, and validation of the novel convolutional neural network model. Finally, Section 5 presents the comparative results of road pothole detection using the developed CNN model, validated through test outcomes and field studies.

## 2. Literature Review

Vibration-based approaches used to detect road potholes rely on evaluating vibrations obtained through motion sensors (accelerometer, gyroscope, etc.) on the road surface. A vehicle behaves differently when encountering road irregularities such as potholes, cracks, or speed bumps, and this behavior can be captured through vibrations. In studies conducted using mobile devices, road surface irregularities are commonly detected from road vibration data using the threshold method. Detection is based on the road vibration data exceeding a predefined threshold value, indicating surface irregularities. However, to minimize the impact of varying environmental conditions, dynamic thresholding is preferred over a fixed threshold approach. For road surface monitoring using dynamic thresholding, a Gaussian-model-based mining algorithm was proposed [29]. Various detection components utilizing smartphone sensors, including accelerometers, microphones, GSM radios, and/or GPS sensors focus on detecting potholes, speed bumps, braking, and honking. Pothole detection primarily focuses on accelerometer data processing, while some studies have used both accelerometer and gyroscope data, including the vehicle’s average speed, to obtain road roughness using the fast Fourier transform (FFT) method [30]. Another approach involves deriving the parameters of the International Roughness Index (IRI) model from GPS and accelerometer data [31]. A system was proposed that verifies road anomalies using smartphone sensor data against vehicle sensor data, classifying them based on their features [32]. Additionally, a system that uses both smartphone sensors and a gyroscope/accelerometer-equipped wireless controller for z-axis algorithms was suggested [33]. For nearly real-time detection and classification of road surface abnormalities, a hybrid method combining threshold-based and ML approaches was recommended [34].

Another widely used method for detecting road defects is machine learning. To allow computers to classify road vibration data, a model must be trained on a prepared dataset. This method enables the classification of road vibrations to identify potholes [21]. Using smartphone sensors, an SVM algorithm was proposed to eliminate speed, gradient, and drift effects from sensor signals and classify road segments [35]. Three-axis accelerometers and gyroscopes in smartphones were used with a decision tree classifier to classify road segments and create models [36]. An early warning system was suggested that uses an SVM algorithm to detect both speed bumps and poor road conditions [37]. For road surface classification, a comparison was made between an SVM, hidden Markov model (HMM), and residual network (ResNet), with ResNet performing the best [38]. Pothole classification was achieved with high accuracy using SVM, and a k-means algorithm was used to distinguish between potholes and speed bumps [39]. An algorithm for road surface recognition using a smartphone acceleration sensor was proposed, optimized by a fuzzy logic inference machine and a Gaussian background model (GBM) [40]. A random forest method was suggested for pothole classification based on features extracted from acceleration signals obtained from smartphones [41]. Hybrid approaches combining threshold-based and ML methods have also been employed [34].

Different studies in the literature also exist where road defects have been detected using machine learning without the use of smartphones. Using an Internet of Things (IoT) sensor, various machine learning models (logistic regression (LR), SVM, k-nearest neighbors (KNN), naive Bayes (NB), decision tree (DT), random forest (RF), and ensemble voting) were compared in terms of their performance under different parameters, and it was determined that random forest was the best model for pothole detection [42]. The use of ML techniques and an IoT platform was recommended for the detection of speed bumps, humps, and potholes [43].

In addition to threshold- and machine-learning-based methods, dynamic time warping has been used to detect road defects using smartphone sensors [24]. A system was proposed that provides real-time alerts to drivers using FastDTW and SVM to detect driving events and road abnormalities [44]. For the detection of potholes on road surfaces, a DTW-based separation algorithm for road vibration signals with higher sensitivity was suggested [45]. SVM, HMM, and residual networks (ResNet) were compared for road pavement and non-pavement classification, while KNN and DTW methods were evaluated for anomaly detection [38]. Since this study uses the ResNet method, it features a deep-neural-network-based approach.

A system was proposed for vehicle tracking, heterogeneous human activity recognition, and biometric motion analysis using motion sensors with smartphones based on the Unified Deep Learning framework, which combines convolutional and recurrent neural networks [28]. While the study did not focus on road surface issues, it compared the results of the DeepSense framework for three different applications based on data collected through mobile device sensors over time. Another study referencing this approach proposed a DeepSense framework for road surface recognition using time series data collected with a smartphone. Additionally, the method’s performance was demonstrated to be superior when compared to CNN, NN, SVM, and random forest (RF) classifiers [27]. To improve the accuracy of human activity recognition, a CNN deep learning model was created and recommended by comparing CNN and RNN methods [46].

In a study focused on examining the convolutional kernel size, an original convolutional neural network architecture was proposed, aiming to discover patterns of accelerometer signals for human activities in each axis within the layers that constitute the network [47]. A novel approach for organizing time series data from smartphone sensor signals for human activity recognition (HAR) was recommended, using a CNN-based classifier [48]. For HAR, both 1D and 2D CNNs were used with inertial signals, and their performances were compared with each other and with SVM, KNN, and MLP methods [49]. A unique shallow CNN with a C3 block was suggested for sensor-based HAR, where all channels in the same layer have extensive interaction to capture distinctive features in raw sensor data [50]. An augmented multichannel convolutional neural network (AMC-CNN) model was proposed to better explore feature patterns in time series and enhance the capability of HAR [51]. To improve the accuracy of human activity recognition (HAR), a novel approach called convolution ternary (HAR-CT) was proposed, which enhances a CNN using the trained ternary quantization (TTQ) approach [52].

For sensor-based human activity recognition (HAR), a multilayer hybrid architecture was designed using CNNs and long short-term memory (LSTM) [53]. Human activity predictions using smartphone accelerometers were compared to results obtained using the LSTM technique [54].

## 3. Methodology and Experimental Design

**Figure 1:**
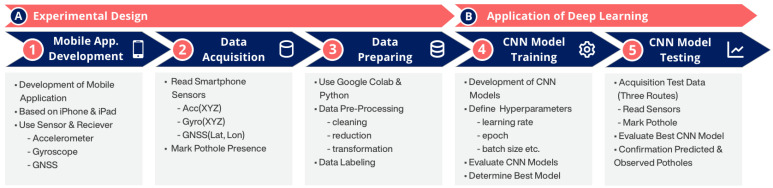
Workflow of the proposed methodology.

Road vibration data were obtained using smartphone sensors for the purpose of identifying road potholes through deep learning. The process of data collection, preprocessing, the creation of the deep learning model, and the detection of road potholes were followed sequentially. To ensure the continuous execution of these processes, an original methodology was developed in this study (Figure 1). The proposed methodology consists of two main steps: (A) conducting the experimental design, and (B) implementing deep learning. To make the methodology more understandable, the application of these two fundamental steps will be addressed in five stages. These stages are as follows: (1) development of the mobile application, (2) collection of road vibration data, (3) preparation of road vibration data through preprocessing, (4) development and validation of the convolutional neural network model, and (5) prediction of road potholes using the CNN model and field validation. While the first two stages form the experimental design (A), the steps related to the implementation of deep learning (B) will be examined under separate headings.

### 3.1. Experimental Design

#### 3.1.1. Development of Mobile Application

**Figure 2:**
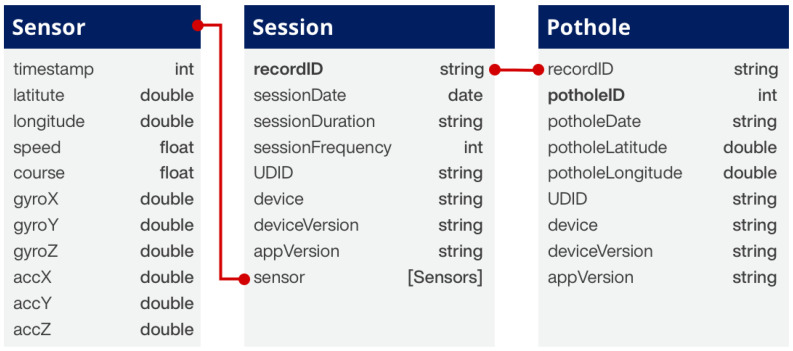
Road vibration data structure.

In the initial stage of the experimental design, which is the first phase of the proposed methodology, decisions were made regarding the functions of the smartphone application to collect road vibration data, the determination of the sensors to be used, and the creation of a database. A mobile application was developed for collecting road pothole vibration data, compatible with the Apple iOS platform and usable with iPhone and iPad devices. In this study, an Apple iPhone 7 device, manufactured by Apple Inc. in California, USA, with Apple A10 Fusion processor and 2 GB LPDDR4 RAM was used for data collection. The sensors used for road pothole detection in smartphones included a 3-axis accelerometer, gyroscope, A-GPS, GLONASS-enabled GNSS, and network. While the 3-axis accelerometer and gyroscope were used to detect road vibrations, the GNSS receiver ensured the recording of collected road vibration data as coordinates and the identification of pothole locations. Internet (network) connection on mobile devices enhanced location accuracy and enabled dynamic map loading. Since the vibration data from these sensors were continuously recorded, all data were stored in a local database within the mobile device. For this purpose, the Realm database [55] was used, which is advantageous in terms of performance compared to many other databases (Sqlite, CoreData, etc.). In the created database, three tables, named Sensor, Pothole, and Session, were designed to store road vibration data obtained from sensors, pothole information, and session data (Figure 2). The developed mobile application was designed to have functions for real-time collection of road vibration data, marking and saving potholes, monitoring and transferring all collected data types, and adjusting parameters related to the data collection process.

#### 3.1.2. Data Acquisition

Three significant factors, such as environmental conditions, including weather, road conditions, and vehicle operation parameters, like tire pressure and vehicle speed, can be considered in the collection of road data. In this study, during the data acquisition (first phase) and model testing (second phase), the environmental conditions remained consistent with a dry day, on a dry asphalt road, and a maximum vehicle speed of 50 km/h, with the same vehicle driver. Both the training and testing of the CNN model were conducted under these environmental conditions.

**Figure 3:**
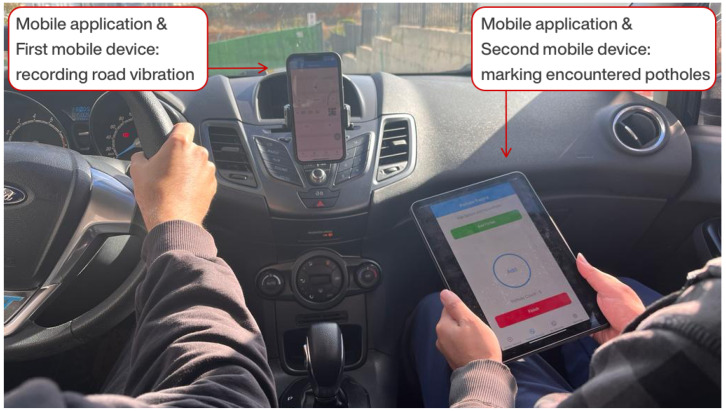
Data acquisition process in the vehicle using smartphone sensors and mobile application.

Using the developed mobile application, road vibration data and road potholes were detected and recorded under unique sessions along a predefined route using a Ford Fiesta vehicle (Figure 3). Two smart mobile devices were used during the process of collecting road data: one fixed in the vehicle, and the other handheld by the driver’s assistant. The two mobile devices were paired via a QR code through the mobile application, enabling data synchronization and time alignment. The operations performed to collect road data and the functions of the developed mobile application during the process of data collection are detailed below.

**Figure 4:**
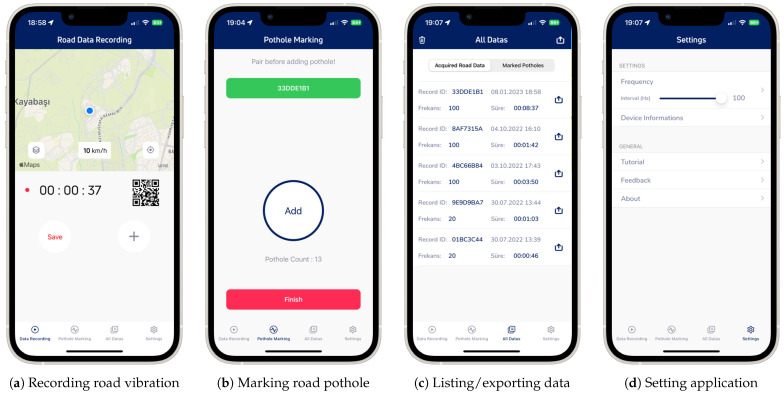
Mobile application interfaces.

The operations conducted to collect road vibration data and the functions of the developed mobile application are as follows (Figure 4):

- Acquisition of road vibration data: Smartphone sensor data were recorded at the desired frequency, and the route was tracked on a map during recording, ensuring equal time intervals for data collection. This step was automated with the first mobile device fixed in the vehicle (Figure 4a).
- Marking road potholes: Initially, the smartphone recording the road vibration data was paired with a QR code. Then, while the vehicle was in motion and passed over road potholes, the driver’s assistant manually marked the potholes to indicate their locations. This step was performed using the second mobile device handheld by the driver’s assistant (Figure 4b).
- Viewing/listing/exporting recorded sensor and marked road pothole data: Data recorded on both devices were exported in CSV format (Figure 4c).
- Monitoring/specifying/modifying application features: The application included features such as adjusting data collection frequency, device information, etc. (Figure 4d).

### 3.2. Data Acquisition and Preparing

When considering the data collection phase, Google Colab [56] was chosen due to its advantages in organizing and processing data in deep learning. Python, the most widely used language in both academic and commercial applications, was employed [57]. Along the designated route, road vibration and pothole data were collected in six different sessions, with approximately 100 data points per second (frequency = 100 Hz), totaling about 2 h in length and 246 MB in size. The road vibration data include attributes such as Timestamp, Latitude, Longitude, Acceleration (Acc(xyz)), and Angular Velocity (Gyro(xyz)), while the pothole data include attributes such as ‘PotholeID’, ‘PotholeLatitude’, and ‘PotholeLongitude’. Two separate database tables, one for road vibration and one for pothole data, were created. To streamline the processing time, unnecessary features were removed from the data that would not be used in the deep learning phase. During the data preparation phase, road vibration and pothole data were organized using different methods.

**Figure 5:**
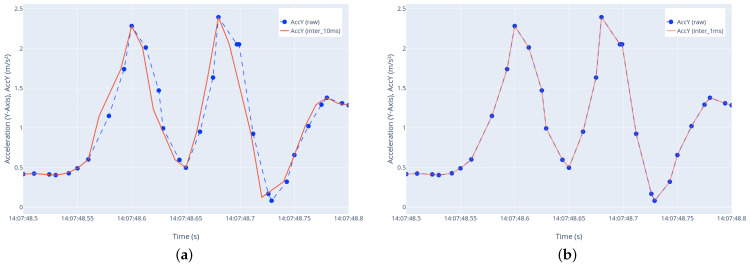
Road vibration data processing. (**a**) Nonuniformly spaced raw data component (85–90 data/s) and uniformly interpolated but lossy data component (100 data/s); (**b**) nonuniformly spaced raw data component (85–90 data/s) and uniformly interpolated final data component (1000 data/s).

Although the smartphone sensor was set to a 100 Hz frequency via the mobile application, there was approximately a 10–15% deviation in the data resolution. This indicates that the collected data were not uniformly spaced over time, suggesting a nonuniform or nonhomogeneous distribution within a unit of time. These raw data, which have a resolution of 85–90 data/s and uniform time intervals, were interpolated to have uniform time intervals at 100 data/s but with some data loss. When these interpolated data were used, small changes in the data curve were observed. To eliminate these discrepancies and increase the resolution to 1000 data/s, the vibration data were subjected to interpolation (Figure 5). As a result, the data had 1000 data/s resolution, equal time intervals (10 ms), and lossless. Different resolutions of these data are compared in Figure 5 for clarity, and for illustrative purposes, only one component, namely, Acc(y), from six road vibration data components is taken as an example. Although this results in an increase in data size, transforming the data into pixel-based images enables the training of the CNN model.

**Figure 6:**
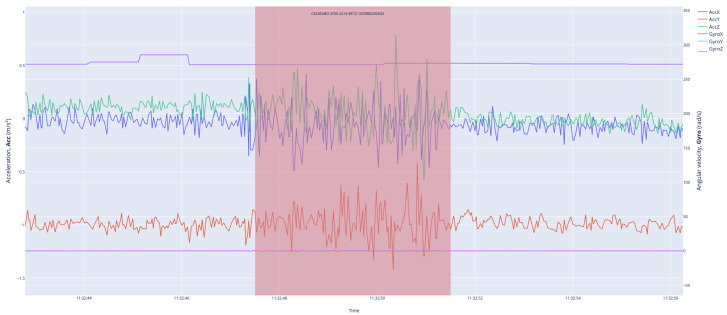
Road vibration data (acceleration Acc(xyz) and angular velocity Gyro(xyz) signals) and pothole region (red area).

The term ‘road pothole data’ differs in meaning from ‘road vibration data’. ‘Road vibration data’ consist of analog signals obtained from smartphone sensors, while ‘road pothole data’ is solely a digital signal indicating the presence of a pothole, meaning ‘there is a pothole here’. Therefore, the second type of data will be treated as ‘marked road pothole data’. The marked pothole data were organized and merged with vibration data without temporal shifts using the QR code method, creating a binary dataset. To convey the characteristics of potholes at different periods accurately to the deep learning stage, the digital signal defining marked pothole data was not represented as an instantaneous signal but, rather, as a periodic step function. By setting the pothole period to 4 s during the examination of the binary dataset, all marked potholes on the road could be transferred to the next stage without loss. The road vibration data, consisting of 3-axis acceleration Acc(xyz) and 3-axis angular velocity Gyro(xyz) signals, and the 4 s pothole region (red area) are shown to be matched (Figure 6). Thus, within the binary dataset, the road pothole region is labeled as ‘Pothole’, while the other regions are labeled as ‘Non-Pothole’, making the road vibration and pothole data ready for deep learning.

## 4. Training and Validating of Convolutional Neural Network Model

**Figure 7:**
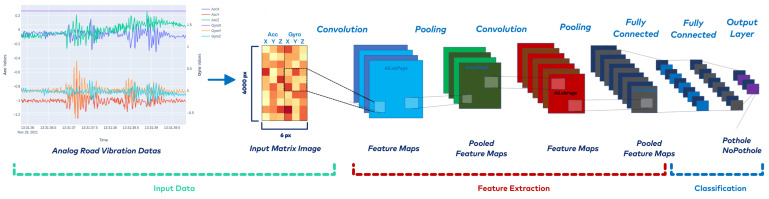
Convolutional neural network detecting road potholes.

Convolutional neural networks, a specialized type of ANN, are one of the most popular and successful approaches for classification and pattern recognition [58]. Unlike traditional machine learning methods, CNNs automatically extract visual features by sliding locally trained filters over input images [58]. The CNN architecture comprises input, convolutional, pooling, fully connected layers, and classified output layers. Convolution and pooling layers perform feature extraction, while the fully connected layer is responsible for classification [59]. In this study, original multicomponent analog road vibration data were transformed into an input visual matrix for the CNN model, as seen in Figure 7. Thus, the road vibration data consisting of 3-component Acc(xyz) and Gyro(xyz) were converted from time-variable analog signals to pixel-based (6×4000 pixels) images. Transforming analog road vibration data into the CNN model is a unique feature of this study.

### 4.1. Model Training and Validating

**Figure 8:**
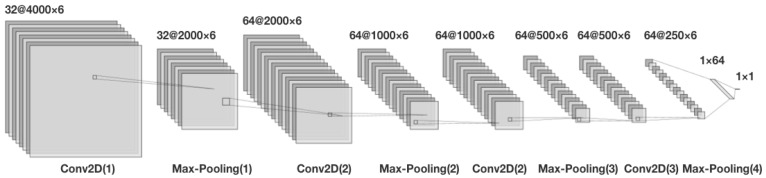
Convolutional neural network model layers and parameters for Model 5.

Different models with varying numbers of layers and parameter values were developed to detect road potholes using a CNN-based approach in the study (Table 1). Initially, a simple model with fewer layers and parameters but good results (Model 1) was developed to serve as the basis for complex models. The number and sizes of layers were gradually adjusted to obtain more advanced models (Models 2–5). Each model was first improved individually, and then a higher-level model with more layers and parameters was developed. Among these models, Model 5, with its layer sizes and parameters detailed in Figure 8, exhibited the highest performance. Additionally, the total number of parameters of the convolutional neural network layers according to the models is given in Table 2. It is worth noting that Model 5 had a relatively low number of parameters despite its high number of layers.

As can be seen from the evaluations above, hyperparameters such as the number of epochs, batch size, learning rate, optimization function, activation function, and train–validation data distribution variables were determined as hyperparameters in the convolutional neural network model. A total of 10 experiments were conducted during the training phase by changing the hyperparameter values differently in the five CNN models (Table 3). It can also be observed from this table which experiment corresponds to which model. Data were divided into two different datasets (432 and 1132 samples) for use in the training phase. It was observed that the size of the data in the dataset affects both the processing time and performance, and as the number of layers of the model increased, the 1132-sample dataset was used to achieve high performance. In addition, these datasets were divided into two for training and validation processes, and two different training/validation ratios (80/20 and 70/30) were used.

By examining the accuracy and loss curves in the experiments, hyperparameters were adjusted to avoid ‘overfitting’ or ‘underfitting’. In experiments based on sub-models (Exp No: 1–3), the batch size was generally 32, which yielded good results except for one exception (Exp No: 5). However, in experiments based on upper models, this value was mostly 64 (Exp No: 7–9), and in the last experiment (Exp. No: 10), it was 128, which resulted in higher performance. Although the Adam and SGD optimization functions were used in only one or two experiments, it was found that the RMSprop optimization function yielded the best results with fewer epochs. When the RMSprop optimization function was used, a learning rate of 0.01, 0.001, and 0.0005 provided the best results in those experiments. However, in experiments based on high-layer models (Exp No: 9–10), learning rates of 0.001 and 0.0005 achieved the best results.

**Figure 9:**
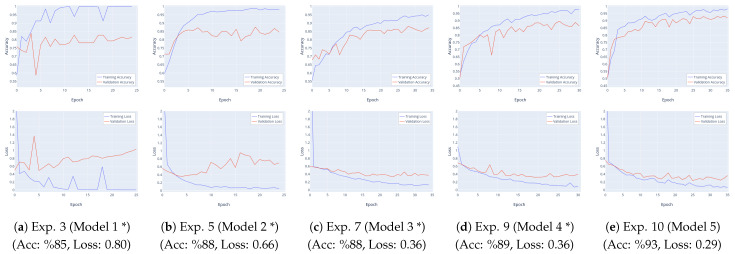
Accuracy and loss variation curves of experiments (for training and validation).

The results corresponding to the hyperparameter values of the experiments listed in Table 3 for training (train) and validation are provided in Table 4. The models marked with (*) in Table 4 and the experiments based on them (Exp. No: 3, 5, 7, 9, 10) are shown with both training and validation accuracy and loss change curves in Figure 9. Considering these results, the previous evaluations will be expanded upon with additional details.

In experiments based on Model 1 (Exp. No: 1–3), training was conducted using the Adam, SGD, and RMSprop optimization algorithms. Despite achieving a high accuracy rate, these experiments resulted in increased loss values, indicating overfitting. After numerous experiments, it was determined that the RMSprop optimization function provided the best results with fewer epochs. Subsequent training sessions used the RMSprop optimization algorithm based on this experience. In Experiment 2, based on Model 2, a dropout layer was added to reduce overfitting. With the same data size, validation accuracy reached up to 89.66%. However, in order to decrease the loss value and further improve performance, data augmentation was applied, increasing the dataset size to 1132 samples. Nevertheless, the performance of Experiment 5 did not improve, and validation accuracy decreased, indicating overfitting.

In experiments based on Model 3, the number of convolutional layers and dropout rates were increased, and a regularization term was added in an attempt to prevent overfitting and enhance performance. Experiment 6 achieved a performance of 87–88% accuracy, maintaining the previous performance level while mitigating overfitting. Upon realizing that increasing the number of layers did not have a direct positive effect on performance and that the training time increased due to an increase in parameters, a pooling layer was added to reduce the number of calculated parameters and computation costs. Experiments based on Model 4 and 5 were conducted by adding a pooling layer (refer to Table 1). Additionally, the data distribution for training/validation was changed to 70–30 instead of 80–20 (refer to Table 3).

In Experiment 9, during training, a validation accuracy of approximately 89% and a loss value of 0.3575 were achieved. In Experiment 10, based on Model 5, by increasing the kernel size of the convolutional layer, the highest validation accuracy of 93.24% and the lowest loss value of 0.2948 were achieved. As a result, the experiment based on Model 5, denoted as Experiment 10, with the highest performance rate, will be referred to as the ‘Proposed Model’ (hyperparameters used in Experiment 10) to adhere to the nomenclature in the literature, especially since the term ‘model’ is used in the title.

### 4.2. Model Testing

In the test dataset, road potholes were denoted as ‘Predicted Pothole’ (‘PredPothole’) and enumerated with ‘PotholeNo’. The responses provided by the recommended CNN model for each route were obtained as predicted values (pv) within the range of ‘0’ to ‘1’. Along each route, if the predicted value returned ‘1’, it unequivocally corresponded to ‘Pothole’, and if it returned ‘0’, it signified ‘Not Pothole’. Consequently, road potholes were identified and labeled as either ‘Pothole’ or ‘Not Pothole’, depending on whether the predicted value approached ‘1’ or ‘0’, respectively, when the predicted value was within the range ‘0’ to ‘1’. When the predicted value fell within this range, and the threshold value was set at ‘0.5’, it was anticipated that the closer the predicted value was to ‘1’, the more likely it would be to indicate ‘Pothole’, while as it diverged, it would indicate ‘Not Pothole’. For ‘PredPotholes’ corresponding to predicted values that fell below the accepted threshold value of ‘0.5’ and for values within the condition of 0.5⩽pv⩽1, they were automatically labeled as ‘Not Pothole’ and ‘Maybe Pothole’, respectively. The ‘Maybe Pothole’ label was temporarily assigned as interim labeling, and potholes exhibiting this characteristic will be physically examined in the field, ultimately being labeled as either ‘Pothole’ or ‘Not Pothole’. Consequently, the proposed CNN model, utilizing road vibration data collected as input via a mobile device within the vehicle, is capable of detecting and labeling all potholes along the road route during any given journey.

Secondly, road potholes identified using the proposed CNN model were further verified through fieldwork, and they were labeled as ‘Pothole’, ‘Not Pothole’, or ‘Maybe Pothole’. The predicted value (pv) falls within the range of 0.5⩽pv⩽1 for ‘Maybe Pothole’ labeled ‘PotholeNo’. These were meticulously examined in the field, and a threshold value of ‘0.95’ was determined to distinguish between ‘Maybe Pothole’ and ‘Pothole’. Consequently, ‘PotholeNo’s within the range of 0.5⩽pv<0.95 were mostly categorized as ‘Not Pothole’, with a few exceptions, such as those related to pedestrian crossing lines, etc. Hence, they were labeled as ‘Not Pothole’. On the other hand, ‘PotholeNo’s with predicted values in the range of 0.95⩽pv⩽1.0 were categorized as ‘pothole’, indicating that they were, indeed, potholes.

## 5. Results and Discussion

### 5.1. Predicted and Observed Results

**Figure 10:**
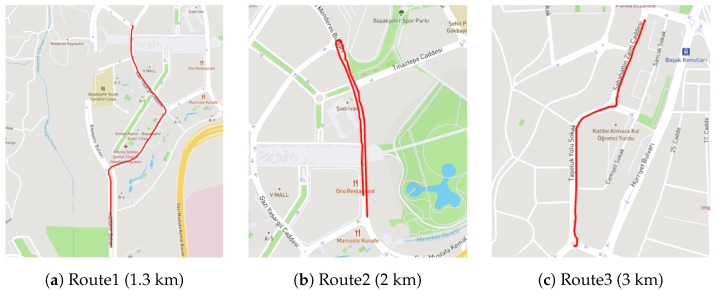
Routes defined for testing and field validation.

In order to accurately conduct the testing of the proposed CNN model, entirely new test road routes were determined (Figure 10). To assess the success of the test results most accurately, a two-stage validation process was implemented. In the first stage, road potholes on the routes were classified based on the labeled results produced by the CNN model, while in the second stage, road potholes were identified through field observations and detections along the route. A total of 187 data points were collected from the three specified test routes for the testing of the CNN model, and the results obtained from the model were validated through fieldwork.

No detailed examination was conducted for the data labeled as ‘Not Pothole’, while for other data, two types of labeling were determined based on the prediction results obtained from the two-stage validation and testing process: (A) those labeled as ‘Pothole’ for prediction values within the range 0.95⩽pv⩽1.0, and (B) those labeled as ‘Not Pothole’ for prediction values within the range 0.5⩽pv<0.95. Furthermore, new situations and detected pothole values were determined through field inspections: (C) observed potholes through fieldwork, (D) observed bumps through fieldwork, and (E) the accuracy rates of potholes predicted through the two-stage process, which are considered as false potholes (Table 5).

Table 5 presents the potholes predicted by the proposed model, potholes observed in the field, and the prediction accuracy of the model based on three test routes. The potholes predicted by the model (A) were confirmed by individual field observations (C). Some of the differences between them are due to the identification of bumps observed in the field (D) and erroneous predictions (E). The accuracy of the model in prediction is approximately in the range of 80–90% for all three routes, calculated using the number of potholes confirmed in the field (C).

This study aimed to detect road potholes and, under the definition of ‘pothole’, it included road irregularities such as cracks, fissures, asphalt sinkholes, and manhole cover sinkholes, while excluding speed bumps. When evaluated within this framework, it was observed that the model also detected some speed bumps as potholes. Field inspections confirmed that some of these speed bumps have effects on vehicles similar to potholes, leading the developed model to perceive them as potholes. The values obtained from three test routes (D) are presented in Table 5. In addition, very few ‘Potholes’ labeled as such did not turn out to be any potholes or speed bumps in their field observations (E).

**Figure 11:**
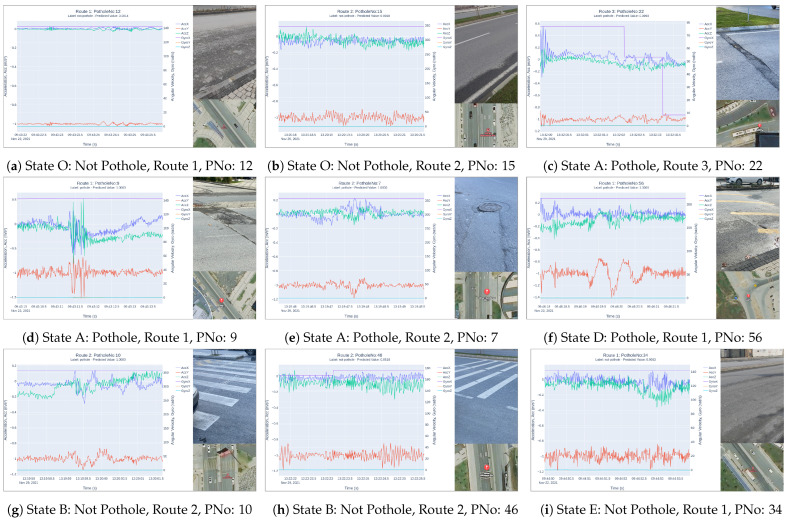
Vibration data, pothole imagery, and location for major pothole states.

In field observations and examinations, it was determined that some of the indexes labeled as ‘Potholes’ were actually not potholes but, rather, speed bumps. Since the same situation was observed and confirmed in all three routes, the number of speed bumps was added to Table 5. When the results obtained from the CNN model’s prediction values and field inspections are considered, it can be seen that the results can be classified into six different situations. For all these situations, the model’s road graphics and images obtained from field inspections are presented in Figure 11 based on the route and ‘PotholeNo’.

- Situation O: Prediction value: 0⩽pv<0.5, labeled as ‘Not Pothole’, and field observations confirmed the absence of potholes.
- Situation A (C): Prediction value: 0.95⩽pv⩽1.0, labeled as ‘Pothole’, and field observations confirmed the presence of potholes.
- Situation A (C): Prediction value: 0.95⩽pv⩽1.0, labeled as ‘Pothole’, and field observations confirmed the presence of manhole covers with a pothole appearance.
- Situation D: Prediction value: 0.95⩽pv⩽1.0, labeled as ‘Pothole’, and field observations revealed speed bumps instead of potholes.
- Situation B: Prediction value: 0.5⩽pv<0.95, labeled as ‘Not Pothole’, and field observations confirmed the presence of features such as pedestrian lane markings and road ridges, but no potholes.
- Situation E: Prediction value: 0.5⩽pv<0.95, labeled as ‘Not Pothole’, and field observations confirmed the absence of any potholes.

### 5.2. Discussion

It is evident that deep learning, specifically the CNN technique, can be used effectively in the detection of road potholes based on the proposed methodology and model. Furthermore, observations were made by comparing the detection of road irregularities such as speed bumps, pedestrian lane markings, and others present on the road during fieldwork. Despite the fact that the vibration data collected from the road during the model’s training phase were labeled solely as ‘Pothole’ and ‘Not Pothole’, it was also noted that the model sometimes perceived speed bumps as potholes, particularly in certain cases (Table 5). In other words, some speed bumps on the road were predicted as potholes by the model, albeit at a low rate. Since the study did not set out to identify and classify other road irregularities, including speed bumps, it is concluded that the proposed CNN model is successful in detecting potholes. However, we aim to further develop the proposed method in future studies to include the identification and classification of road irregularities beyond potholes.

The acquisition of the proposed CNN model in this study was based on the training and testing processes conducted under the same environmental conditions (weather, road, and vehicle operation). However, it is considered beneficial to investigate the extent to which the developed CNN model for pothole detection can maintain the same level of success under varying environmental conditions in future studies. Additionally, the development of a new CNN model to include different environmental conditions can be revealed in future research.

In contrast to image-based approaches that focus on road surface images, this study proposed a vibration-based method for collecting road data, specifically emphasizing only the road vibrations collected from the contact area where the vehicle’s tires touch the road surface. The CNN model, which detects potholes by learning from road vibrations, will not be able to detect road potholes when the vehicle’s tires do not make contact with them. Hence, it is considered that using the proposed method in a crowd-based mobile application can ensure that the entire road area comes into contact with the tires.

## 6. Conclusions

In the context of road anomaly detection, studies generally prefer vision-based and vibration-based approaches. In the former, both ML techniques and CNNs, DL methods, are commonly employed, while the latter predominantly relies on thresholding and machine learning methods. Furthermore, when examining studies related to HAR based on vibration-based approaches, it is observed that CNN techniques are extensively utilized in these studies as well [47,48,49,50,51,52].

In this study, all road data were transformed into pixel-based images, enabling the utilization of a convolutional neural network as a deep learning technique for the detection of road potholes. Therefore, this study proposed a novel CNN model. Under the topic of “Road Surface Recognition”, successful outcomes were achieved by using vibration-based road data collected via smartphone sensors, particularly in the detection of road potholes.

Within the scope of the proposed method, vibration data from a three-component sensor integrated with a smartphone (Acc(xyz) and Gyro(xyz)) were converted from analog signals into pixel-based images. Five different CNN models with varying layer numbers and dimensions were developed, and a total of ten experiments were conducted. The CNN model achieved a maximum accuracy rate of 93.24% and a minimum loss value of 0.2948 during validation.

In this study, the validation of the proposed CNN model on three selected test routes was carried out in two stages, including fieldwork. When the accuracy rate in detecting road potholes on test routes was confirmed through fieldwork, it was observed that the accuracy reached approximately 80–90% on two of the routes. Additionally, the study evaluated each of the six different prediction scenarios separately by considering the fieldwork results in conjunction with the CNN model’s predictions.

## Figures and Tables

**Table 1:** Convolutional neural network models, layers, and parameters.

Model Name	Layer Types	Number of Layers
Model 1	Conv(16,(2,2)) – ReLU Conv(32,(2,2)) – ReLU Flatten - Dense(64) – ReLU – Dense(1) – Sigmoid	5
Model 2	Conv(16,(2,2)) – ReLU – Dropout(0.1) Conv(32,(2,2)) – ReLU – Dropout(0.1) Flatten– Dense(64) – ReLU – Dropout(0.5) – Dense(1) – Sigmoid	5
Model 3	Conv(16,(2,2)) – ReLU – l2(0.0001) – Dropout(0.3) Conv(32,(2,2)) – ReLU – l2(0.0001) – Dropout(0.3) Conv(32,(2,2)) – ReLU – l2(0.0001) – Dropout(0.3) Flatten – Dense(64) – ReLU – Dropout(0.5) – Dense(1) – Sigmoid	6
Model 4	Conv(32,(3,3)) – ReLU – l2(0.0001) – MaxPooling – Dropout(0.3) Conv(64,(3,3)) – ReLU – l2(0.0001) – MaxPooling – Dropout(0.3) Conv(64,(3,3)) – ReLU – l2(0.0001) – MaxPooling – Dropout(0.3) Conv(64,(3,3)) – ReLU – l2(0.0001) – MaxPooling – Dropout(0.3) Flatten – Dense(64) – ReLU – Dropout(0.5) – Dense(1) – Sigmoid	11
Model 5	Conv(32,(5,5)) – ReLU – l2(0.0001) – MaxPooling – Dropout(0.3) Conv(64,(5,5)) – ReLU – l2(0.0001) – MaxPooling – Dropout(0.3) Conv(64,(5,5)) – ReLU – l2(0.0001) – MaxPooling – Dropout(0.3) Conv(64,(5,5)) – ReLU – l2(0.0001) – MaxPooling – Dropout(0.3) Flatten – Dense(64) – ReLU – Dropout(0.5) – Dense(1) – Sigmoid	11

**Table 2:** Total number of parameters of convolutional neural network models.

Model Name	Parameter Count
Model 1	32,753,905
Model 2	32,753,905
Model 3	24,563,985
Model 4	6,236,801
Model 5	6,401,153

**Table 3:** Hyperparameter values of experiments.

Experiment Number	Model Name	Data Count	Data Split	Batch Size	Optimization Function	Learning Rate	Epock Count
1	Model 1	432	80/20	32	Adam	0.001	28
2	Model 1	432	80/20	32	SGD	0.001	30
3	Model 1	432	80/20	32	RMSprop	0.001	24
4	Model 2	432	80/20	32	RMSprop	0.01	27
5	Model 2	1132	80/20	16	RMSprop	0.001	25
6	Model 3	1132	80/20	32	RMSprop	0.001	25
7	Model 3	1132	80/20	64	RMSprop	0.001	35
8	Model 4	1132	70/30	64	RMSprop	0.0005	31
9	Model 4	1132	70/30	64	Adam	0.0005	35
10	Model 5	1132	70/30	128	RMSprop	0.001	36

**Table 4:** Training and validation results of experiments.

Exp. Number	Model Name	Training		Validation	
		**Accuracy (%)**	**Loss**	**Accuracy (%)**	**Loss**
1	Model 1	98.93	0.0589	86.21	0.4333
2	Model 1	94.92	0.1981	81.66	0.4707
3	Model 1 *	100.00	0.0138	85.06	0.8016
4	Model 2	94.17	0.1513	89.66	0.5036
5	Model 2 *	98.94	0.0710	87.67	0.6554
6	Model 3	92.00	0.1569	87.22	0.3349
7	Model 3 *	93.90	0.1282	88.11	0.3639
8	Model 4	96.85	0.1029	90.00	0.3614
9	Model 4 *	97.24	0.1183	89.12	0.3575
**10**	Model 5 *	**97.08**	**0.0924**	**93.24**	**0.2948**

* It was chosen because it corresponds to the experiment that yielded the most successful result within the model with the same name.

**Table 5:** Predicted and field-observed results of pothole detection based on test routes.

Route Name	Predicted by Model		Field-Observed			Prediction Acc.	
	Pothole	Not Pothole	Pothole	Bump	Conflict	Ratio	Pct (%)
	(0.95 ≤ *pv* ≤ 1)	(0.5 ≤ *pv* < 0.95)					
State	A	B	C	D	E	(C+D)/A	
Route1	29	9	22	3	4	25/29	86.2
Route2	24	3	15	6	1	21/24	87.5
Route3	15	4	8	4	2	12/15	80.0

*pv*: Predicted value.

## Data Availability

Not applicable.

## Abbreviations

The following abbreviations are used in this manuscript:

MDPI	Multidisciplinary Digital Publishing Institute
A-GPS	Assisted Global Positioning System
GLONASS	Global Orbiting Navigation Satellite System
GNSS	Global Navigation Satellite Systems
CSV	Comma Separated Values
CONV	Convolutional
ReLU	Rectified Linear Unit
SGD	Stochastic gradient descent
RMSprob	Root mean square error probability
PCT	Percentage
ACC	Accuracy
